# Frailty: A global measure of the multisystem impact of COPD

**DOI:** 10.1177/1479972317752763

**Published:** 2018-01-16

**Authors:** Nichola S Gale, Ali M Albarrati, Margaret M Munnery, Ruth E Hubbard, Ruth Tal-Singer, John R Cockcroft, Dennis J Shale

**Affiliations:** 1 School of Healthcare Sciences, Cardiff University, University Hospital of Wales, Cardiff, UK; 2 College of Applied Medical Sciences, King Saud University, Riyadh, Saudi Arabia; 3 School of Health Sciences, Cardiff Metropolitan University, Llandaff Campus, Western Avenue, Cardiff, UK; 4 Centre for Research in Geriatric Medicine, The University of Queensland, Brisbane, Australia; 5 GlaxoSmithKline R&D, King of Prussia, PA, USA

**Keywords:** Aging, comorbidities, COPD, frailty, systemic

## Abstract

Chronic obstructive pulmonary disease (COPD) is a multisystem disease that resembles the accumulation of multiple impairments seen in aging. A comprehensive geriatric assessment (CGA) captures multisystem deficits, from which a frailty index (FI) can be derived. We hypothesized that patients with COPD would be frailer than a comparator group free from respiratory disease. In this cross-sectional analysis, the CGA questionnaire was completed and used to derive an FI in 520 patients diagnosed with COPD and 150 comparators. All subjects were assessed for lung function, body composition, 6-minute walking distance (6MWD), and handgrip strength. Patients completed validated questionnaires on health-related quality of life and respiratory symptoms. Patients and comparators were similar in age, gender, and body mass index, but patients had a greater mean ± SD FI 0.16 ± 0.08 than comparators 0.05 ± 0.03. In patients, a stepwise linear regression 6MWD (*β* = −0.43), number of comorbidities (*β* = −0.38), handgrip (*β* = −0.11), and number of exacerbations (*β* = 0.11) were predictors of frailty (all *p* < 0.01). This large study suggests patients with COPD are frailer than comparators. The FI derived from the CGA captures the deterioration of multiple systems in COPD and provides an overview of impairments, which may identify individuals at increased risk of morbidity and mortality in COPD.

## Introduction

Chronic obstructive pulmonary disease (COPD) is one of the few major chronic conditions increasing in prevalence. The defining feature of COPD is impaired lung function, but extra-pulmonary comorbidities contribute to reduced health-related quality of life (HRQoL), increased morbidity, and mortality risk.^1,2^ Quantifying multimorbidity is challenging as current routine clinical assessments do not take account of comorbidities. However, a scoring system based on body mass index (BMI), airflow obstruction, dyspnea and exercise tolerance (BODE index) along with comorbidities predicted mortality risk over a 4-year period.^1^

Comorbidities represent deficits in individual physiological systems and resemble the accumulation of deficits in natural aging, which may result in frailty.^3,4^ Frailty is associated with aging and manifests as failure to respond to external stresses and maintain homeostasis.^5^ Frailty better predicts adverse outcomes than age, independent of coexisting medical conditions.^6^ The similarities between the accumulation of deficits in aging and multiple comorbidities in COPD suggests that frailty is likely to occur in COPD and be related to increased morbidity.^3^ Studies in a range of general populations and COPD patients have produced an inconsistent prevalence of frailty in COPD^7,8^ ranging from 10% frail and 51% intermediate frail, in the Rotterdam study^9^ to 57.8% frail in physician diagnosed COPD,^10^ and 25.6% in patients referred for pulmonary rehabilitation.^8^ A recent study reported 21% frailty in a Japanese COPD cohort.^11^ In addition to various populations studied, there were differences in study design, methods to assess frailty, and lack of appropriate population controls.^12,13^

There are a number of validated tools to assess frailty.^3,12,13^ The key ones in current use include the following. (1) The phenotypic definition based on the presence of ≥3 of five deficits including walking speed, handgrip strength, activity levels, weight loss, and exhaustion,^3^ which has been criticized for not grading or specifying degrees of frailty. (2) To consider frailty as a multidimensional, risk state quantified by the number, rather than the nature of physiological deficits.^3,6,13^ Using a widely validated questionnaire to capture information on health status and function, the comprehensive geriatric assessment (CGA) and a frailty index (FI-CGA) can be derived.^14^ In many populations, this operationalized approach to defining and quantifying frailty has demonstrated that accumulated deficits are a stronger predictor of mortality than chronological age.^13^ In geriatric medicine, the FI-CGA is used clinically to assess the multisystem impacts of aging and indicate premature aging.^6,14^

Although there have been other studies of frailty in COPD, to our knowledge, this is the first to explore and report frailty using the CGA, in a well-characterized population of community-dwelling patients with COPD, along with a group of comparators similar in age and sex from the same community. We hypothesized that frailty occurs in COPD based on the FI-CGA assessment and that patients would be more frail than comparators independent of age, which may indicate an accelerated and premature aging process in COPD.

## Methods

### Study design and participants

This cross-sectional analysis included community-based patients with COPD, confirmed by spirometry at entry, and comparators (current or ex-smokers free from respiratory disease). Participants were drawn from the prospective Assessment of Risk in Chronic Airways Disease Evaluation (ARCADE) study (Clinical Trials.gov, NCT01656421)^15^ and included all participants who completed the CGA. All participants were clinically stable, not having received or oral corticosteroids in the previous 4 weeks prior to recruitment. Exclusions included inflammatory disease such as rheumatoid arthritis, oral maintenance corticosteroids, inflammatory bowel syndrome, and long-term oxygen therapy. The exclusions were to study a sample of patients representative of the general COPD population, without significant impairment due to recent exacerbation or use of long-term oxygen therapy (LTOT).

Participants were recruited from respiratory outpatient clinics, pulmonary rehabilitation, smoking cessation referrals, general practice databases, and previous participants in respiratory research at Cardiff University. Comparators were recruited, based on being a current or past smoker, from primary and smoking cessation clinics, as well as past volunteers in research and partners of patients with COPD, in an attempt to standardize socioeconomic differences. All participants gave written informed consent and the study had approval from the South East Wales Research Ethics Committee.

### Frailty and questionnaires relating to COPD

All participants completed a modified version of the CGA questionnaire,^4^ specific to community-dwelling individuals. The CGA collects data on self-rated health, psychological well-being, mobility, function, nutritional status, medications, and comorbidities that are coded to reflect deficits (Online Supplemental 1). In community-dwelling participants of the Canadian Study of Health and Aging, the FI-CGA was a valid and reliable means of quantifying health status, stratifying patients’ risk of institutionalization and death.^16^ The CGA was modified by removal of the components specific to hospital admission and data were collected by a researcher-administered questionnaire that took less than 10 minutes to complete. The FI-CGA was calculated by dividing the total number of CGA deficits by the maximum possible score of 61.

Patients with COPD completed the St. George’s respiratory questionnaire (SGRQ), a validated disease-specific measure of HRQoL, scores ranging from 0 to 100, with high scores representing worse status,^17^ and the COPD assessment test (CAT), scored 0–40 with high scores indicating more symptoms.^18^ Patients reported the number of respiratory exacerbations (defined as requirement for antibiotic or oral corticosteroid therapy) per year^19^ and the number of previously diagnosed comorbidities in patients and comparators were also self-reported using a standardized questionnaire that was verified by researcher at the clinical assessment. Infrequent exacerbators were defined as <2 in the previous year, while frequent exacerbators as ≥2. Breathlessness was recorded using the modified Medical Research Council (mMRC) dyspnea scale.

### Lung function

All participants performed spirometry (Vitalograph Alpha, Vitalograph Ltd), to determine forced expiratory volume in one second (FEV_1_), forced vital capacity (FVC), and the FEV_1_:FVC ratio. A diagnosis of COPD was confirmed as post bronchodilator spirometry FEV_1_:FVC <0.70.^19^ Patients were classified according to the combined assessment, Global initiative for chronic Obstructive Lung Disease (GOLD) A–D based on the CAT score.^19^

### Body composition and physical performance

Weight and body composition were measured barefoot in lightweight indoor clothing; fat percentage, fat-free mass (FFM), and the BMI (kg/m^2^) were determined using a segmental bioelectrical impedance analyzer (BC418 Tanita Corp, Tokyo). The FFM index (FFMI) was expressed as a height-squared ratio FFMI (kg/m^2^). A stretch resistant tape measure was used to measure waist and hip circumference.

All participants completed the 6-minute walking distance (6MWD) test with pretest resting heart rate and oxygen saturation by pulse oximetry.^20^ Mean maximum handgrip strength, twice with each hand, was determined using a hand dynamometer (Takei, Japan).

### Inflammation

High sensitivity C-reactive protein (HsCRP) and fibrinogen were determined in venous blood by standard procedures (University Hospital of Wales, Biochemistry).

### Data analysis

Data analysis was performed using the Statistical Package for the Social Sciences (SPSS, Chicago, USA), version 18.0. Results are presented as mean ± SD, geometric mean for log_10_ transformed data and median, interquartile range (IQR). Analyses included the independent *t* test, Wilcoxon test, Pearson’s (*r*) or Spearman’s (*r*_s_) correlation, and stepwise multiple regression.

## Results

### Participant characteristics

The FI was greater in the patients 0.16 ± 0.08 than the comparators 0.05 ± 0.03 (*p* < 0.001). Patients also had poorer lung function FEV_1_, FVC and FEV_1_/FVC, greater smoking history, larger waist circumference, lower handgrip, and 6MWD, as well as greater inflammation (HsCRPlog_10_ and fibrinogen). However, patients and comparators were similar in age, gender, BMI, and body composition (Table 1).

**Table 1.** Subject characteristics.^a^

	Comparator (*n* = 150)	COPD (*n* = 520)	*p*	COPD frail (*n* = 143)	COPD non-frail (*n* = 377)	*p*
CGA total	2.25 (1.25–4.0)	8.75 (5.75–12.75)	<0.001	15.25 (13.25–17.5)	6.75 (4.25–9.25)	<0.001
FI	0.05 ± 0.03	0.16 ± 0.08	<0.001	0.26 ± 0.06	0.11 ± 0.05	<0.001
Gender, male:female	76:74	270:250	0.451	61:82	209:168	0.009
Age (years)	65.0 ± 7.4	66.1 ± 7.6	0.109	63.7 ± 8.2	67.1 ± 7.1	<0.001
FEV_1_/FVC (L)	0.78 ± 0.05	0.53 ± 0.11	<0.001	0.52 ± 0.10	0.53 ± 0.11	0.278
FEV_1_ (% predicted)	105 ± 14	58 ± 19	<0.001	53 ± 18	60 ± 19	<0.001
FVC (% predicted)	109 ± 15	87 ± 21	<0.001	81 ± 21	89 ± 20	<0.001
Smoking (pack years)	22 ± 18	41 ± 25	<0.001	42 ± 26	40 ± 25	0.487
BMI (kg/m^2^)	28.1 ± 4.1	28.0 ± 5.5	0.951	29 ± 7	28 ± 5	0.038
Waist circumference (cm)	95 ± 10	100 ± 15.0	0.001	102 ± 16	99 ± 15	0.015
Hip circumference (cm)	105 ± 9	104 ± 11	0.25	106 ± 13	104 ± 11	0.041
Fat%	33.3 ± 7.8	34.1 ± 8.4	0.345	36 ± 10	34 ± 8	0.026
FFMI (kg/m^2^)	18.5 ± 2.3	18.1 ± 2.6	0.097	18.1 ± 2.7	18.1 ± 2.6	0.980
Handgrip (kg)	31.3 ± 10.3	27.1 ± 9.7	<0.001	23.3 ± 9.5	28.5 ± 9.4	<0.001
6MWD (m)	502 ± 85	335 ± 125	<0.001	244 ± 113	366 ± 113	<0.001
Fibrinogen (g/L)^b^	3.08 ± 1.25	3.51 ± 1.31	<0.001	3.6 ± 1.3	3.4 ± 1.3	0.028
HsCRP (mg/ml)^b^	1.76 ± 3.18	3.49 ± 2.89	<0.001	4.1 ± 1.6	3.2 ± 2.8	0.021
mMRC	^d^	2 (1–3)	^d^	3 (2–4)	2 (1–3)	<0.001
SGRQ total^c^	^d^	53 (36–68)	^d^	69 (57–78)	45 (31–58)	<0.001
CAT score^c^	^d^	21 (14–27)	^d^	28 (24–32)	18 (13–23)	<0.001

6MWD: 6-minute walk distance; BMI: body mass index; CAT: COPD assessment test; CGA: comprehensive geriatric assessment; FEV1: forced expiratory volume in 1 second; FFMI: fat-free mass index; FVC: forced vital capacity; HsCRP: high sensitivity C-reactive protein; mMRC: modified Medical Research Council; SGRQ: St George’s respiratory questionnaire; IQR: interquartile range.

^a^All data mean ± SD or median (IQR); *p* < 0.05 significant difference between groups.

^b^Geometric mean.

^c^ *n* = 500.

^d^Not assessed.

Female patients had greater FI 0.17 ± 0.09 than males 0.14 ± 0.09 (*p* < 0.001) and female comparators had greater FI 0.06 ± 0.04 than males 0.04 ±0.03.

### Frailty and characteristics of COPD

Based on the upper 90th centile FI for comparators (0.09) as a cutoff for frailty, 76% of the patients and 13% of comparators were frail. However, using the upper 50% confidence interval of the age adjusted cutoff for frailty from the Survey of Health, Ageing and Retirement in Europe (SHARE) cohort (a large general, unselected, age-related European population), 28% (*n* = 143) of patients and 0% of comparators were frail.

Frail patients (according to SHARE) were younger, had poorer lung function, greater BMI, waist, and fat percentage as well as poorer handgrip and 6MWD compared to non-frail patients. Frail patients also had greater inflammation (interleukin (IL)-6 and HsCRP) and more exacerbations, symptoms (CAT) and poorer quality of life (SGRQ), all *p* < 0.05 (Table 1).

### Medical history

Patients with COPD had more comorbidities and reported more medications than comparators (*p* < 0.05). The most commonly reported comorbidity in both patients and comparators was hypertension and hypercholesterolemia.

Frail patients with COPD had more comorbidities including a greater proportion reporting cardiovascular disorders as well as diabetes, osteoporosis and osteoarthritis, and more medications than non-frail patients with COPD (*p* < 0.05; Table 2). This remained after adjustment of age and the number of comorbidities: frail 0.18 ±0.05 versus non-frail 0.14 ±0.4 (*p* < 0.05). Of the patients, frequent exacerbators (>2/year), *n* = 310, had a greater mean FI 0.19 ± 0.09 than infrequent exacerbations (0–1/year), *n* = 210, 0.15 ± 0.08, and both had a greater FI than that of the comparator group, 0.05 ± 0.04 (*p* < 0.001).

**Table 2.** Comorbidities, exacerbations, and medications in patients and comparators.^a^

	Comparator, *n* = 150	COPD, *n* = 520	*p*	COPD non-frail, 377	COPD frail, 143	*p*
No. of comorbidities	2 (1–3)	3 (2–4)	<0.001	2 (1–3)	1 (0–2)	<0.001
Hypertension	34	245	<0.001	166	79	0.030
Angina	0	61	<0.001	35	26	0.050
Myocardial infarction	0	46	0.002	26	20	0.011
Atrial fibrillation	5	42	0.018	30	12	0.871
Heart failure	0	19	0.025	15	4	0.521
Other heart disease^c^	4	40	0.045	21	20	0.001
Transient ischemic attack	2	36	0.009	18	18	0.002
Hypercholesterolemia	41	237	<0.001	157	80	0.003
Diabetes mellitus	0	67	<0.001	38	29	0.002
Osteoporosis	9	87	0.001	46	41	<0.001
Peripheral vascular disease	3	19	0.317	8	11	0.003
Osteoarthritis	40	177	0.089	113	64	0.001
No. of exacerbations/year∼	^b^	2 (1–3)	^b^	3 (2–3)	1 (1–3)	<0.001
No. of medications	2 (0–3)	5 (3–8)	<0.001	5 (3–7)	8 (5–11)	<0.001
ACE inhibitors	9	115	<0.001	77	38	0.130
Angiotensin receptor blockers	2	44	0.002	31	13	0.749
Calcium channel blockers	8	114	<0.001	78	36	0.267
Beta blockers	5	42	0.047	31	11	0.844
Diuretics	11	109	<0.001	79	30	0.992
Anticoagulants	2	27	0.041	21	6	0.528
Statins	27	192	<0.001	128	64	0.022
Bisphosphonates	5	72	<0.001	37	35	<0.001
Respiratory inhalers^d^	0	419	<0.001	294	120	0.134

ACE: angiotensin-converting enzyme.

^a^All data mean ± SD or median  interquartile range (IQR); *p* < 0.05 significant difference between groups.

^b^Not assessed.

^c^Other heart disease: arrhythmia, enlarged heart, heart murmur, valve disease, vessel disease.

^d^Inhalers: bronchodilators, inhaled corticosteroids, anti-muscarinic, alone or in combination

**Figure 1.**
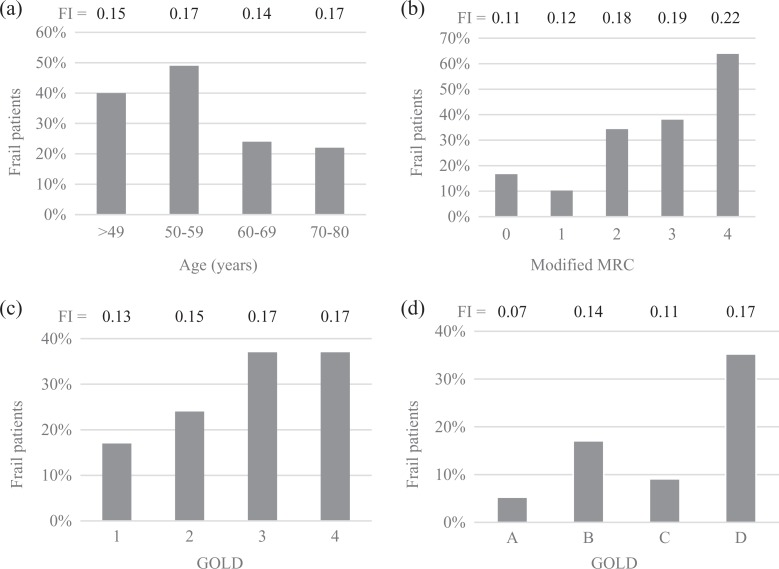
Mean frailty index (FI) and percentage of frail patients classified by (a) age categories, (b) Medical Research Council (MRC) dyspnea score, (c) Global initiative for chronic Obstructive Lung Disease (GOLD) 1–4, and (d) GOLD A–D (using the CAT score).

Using the SHARE cutoff for frailty for each decade,^21^ mean FI and the percentage of frail patients differed across age categories (Figure 1(a)), MRC dyspnea (Figure 1(b)), GOLD 1–4 (Figure 1(c)), and classified according to the GOLD 1–4 and using the combined assessment of COPD A–D using the CAT score (Figure 1(d); all *p* < 0.05). Age and gender did not differ across the classifications and only the number of comorbidities differed across the GOLD categories A–D.

When adjusted statistically for the number of comorbidities, the number of patients classified as frail according to SHARE reduced to *n* = 75. The proportion of frail patients no longer differed across the mMRC, GOLD 1–4, or GOLD A–D categories (*p* > 0.05).

### Relationships between FI and participant characteristics

In COPD, FI was inversely related to FEV_1_%, handgrip, and 6MWD. Measures of body composition (BMI, waist circumference, and fat percentage) and inflammation (HsCRP and fibrinogen) were directly related to FI, along with disease specific questionnaires as well as the number of comorbidities and exacerbations per year (all *p* < 0.01; Table 3).

**Table 3.** Relationship between FI and participant characteristics.^a^

	COPD	*p*	Comparators	*p*
Age	0.16	0.715	0.124	0.132
FEV_1_%	−0.189	<0.001	−0.220	0.007
FEV_1_/FVC	−0.052	0.233	0.120	0.143
Smoking pack years	0.050	0.258	−0.014	0.870
BMI	0.227	<0.001	0.323	<0.001
Waist circumference	0.213	<0.001	0.278	0.001
Fat%	0.218	<0.001	0.366	<0.001
Handgrip	−0.324	<0.001	−0.185	0.024
6MWD	−0.597	<0.001	−0.360	<0.001
HsCRP_log10_	0.158	<0.001	0.294	<0.001
Fibrinogen _log10_	0.181	<0.001	0.200	0.016
No. of comorbidities^b^	0.541	<0.001	0.473	<0.001
No. of exacerbation/year^b^	0.327	<0.001	—	—
Dyspnea (mMRC)^b^	0.466	<0.001	—	—
SGRQ total^b^	0.615	<0.001	—	—
CAT score^b^	0.594	<0.001	—	—

6MWD: 6-minute walk distance; BMI: body mass index; CAT: COPD assessment test; CGA: comprehensive geriatric assessment; FEV1: forced expiratory volume in 1 second; FFMI: fat-free mass index; FVC: forced vital capacity; HsCRP: high sensitivity C-reactive protein; SGRQ: St George’s respiratory questionnaire; FI: frailty index; mMRC: modified Medical Research Council; COPD: chronic obstructive pulmonary disease.

^a^ *p* < 0.05 significant correlation.

^b^Spearman’s rank correlation.

In patients, a stepwise linear regression with the FI as a dependent variable, FEV_1_%, BMI, handgrip, 6MWD, number of exacerbations per year, number of comorbidities and fibrinogen_log10_, as were included as independent variables, based on the results of correlations between CGA and the independent variables. This produced a significant model which explained 51% of the variance of frailty. 6MWD (*β* = −0.43), was the primary predictor, followed by number of comorbidities (*β* = −0.38), handgrip (*β* = −0.11), and number of exacerbations (*β* = 0.11; all *p* < 0.01). In comparators, number of comorbidities (*β* = −0.432), 6MWD (*β* = −2.33), and BMI (*β* = 0.22) explained 38% of the variance of frailty (*p* < 0.01).

In frail patients, the FI was related to BMI (*r* = 0.12), fat mass (*r* = 0.13), waist (*r* = 0.24), HGS (*r* = −0.26), 6MWD (*r* = −0.46), number of exacerbations (*r*_s_ = 0.14), SGRQ (*r*_s_ = 0.48), and CAT (*r*_s_ = 0.45) (all *p* < 0.05), but not inflammation.

## Discussion

In this study, patients with COPD were frailer than a comparator group, similar in age, gender and BMI but free from respiratory disease, and as demonstrated by their greater number of deficits measured as part of the CGA and higher FI score. In our female patients and comparators, the FI was greater than in their counterpart males, a similar relationship to that reported in the general population. The mean FI of females with COPD was greater than the reported FI of 0.15 for women over 65 years of age from general populations.^22–24^

The finding that 28% of our patients were frail based on the SHARE cohort is similar to a recent UK study in patients referred for pulmonary rehabilitation which reported that 26% of their patients were frail using a hybrid of Fried’s criteria.^8^ A recent study reported a prevalence of 21% frailty in patients with COPD using the Kihon Checklist.^11^ These studies are lower than a UK population study where the reported prevalence of frailty was 57.8% in 70 COPD patients diagnosed on the basis of symptoms rather an objective measure of airways obstruction.^10^ Unlike the latter study, our study population was unselected based on physician defined COPD with mandatory demonstration of airways obstruction at the time of entry to the ARCADE study^15^ and hence provided a wider spectrum of disease severity and comorbidities which might account for the variation. Comparisons between this study and other studies^7,25^, are difficult due to different assessments of frailty and older study populations, which may not represent a typical COPD population. We used the validated CGA tool, which allowed our participants to be compared with other populations and has shown to be a predictor of outcomes than chronological age, disability, and the need for health-care support.^6,13^, Based on our own cutoff from frailty from our comparator group, the percentage of frail patients was found high (76%). This may be a result of low levels of frailty in comparators due to rigorous exclusion criteria and self-selection of volunteers for the study.

Using the SHARE cutoff, frail patients had reduced physical capacity, greater systemic inflammation, worse symptoms, and quality of life. Hence, frailty in COPD appears to have impacts and background mechanisms similar to those in natural aging where it is part of the spectrum of multimorbidity. There is likely to be two aspects of COPD interwoven with each other and frailty; the physiological processes of natural aging occurring in all individuals and the impact of COPD and its associated exacerbations and greater comorbidity levels.^26^ Comorbidities in COPD are an important factor in the overall morbidity and mortality^1^ and their interaction may be related to thegreater systemic inflammatory state in aging – inflammaging – and the inflammatory surges during exacerbations superimposed on the background inflammatory state in COPD.^2^ This speculation is supported by the association between frailty and the number of comorbidities and number of exacerbations per year seen in this study. This potentially provides a testable explanation of premature aging in COPD.

Although, frailty in COPD was unrelated to chronological age, the proportion of frail patients differed across the age categories. Interestingly, patients aged 50–59 had greatest FI and the largest proportion of frail patients which could potentially be explained by a survival bias where frail patients fail to reach older age. In addition, frailty differed according to the disease severity classified by GOLD, in mild disease (GOLD 1), 17% of patients were frail increasing to 34% frail in GOLD 3 and 4. Patients in GOLD categories B&D with more symptoms as measured by the CAT score were frailer. These differences did not remain when frailty was adjusted for the number of comorbidities. This suggests that frailty may be as a result of increasing deficits which may occur prematurely even in mild COPD.

Frailty associated with components of body composition, including BMI, fat percentage, and waist circumference. The linear relationship with BMI contrasts with the U-shaped distribution reported in the English Longitudinal Study of Ageing, but may be a consequence of small numbers of individuals with a low BMI.^22^ In both COPD and natural aging, increases in fat and abdominal obesity have been closely linked to impaired physical function.^27^ Increased fat mass and frailty in the older people have also been linked to increased levels of circulating inflammatory biomarkers.^28^ Circulating CRP, an indicator of IL-6 activity, increases with age and has been linked to frailty and truncal obesity, independent of BMI, which may reflect the negative impact of chronic low grade systemic inflammation, a feature of COPD, on physical function and well-being.^29^ Hence, our findings suggest frailty in COPD is a multifactorial function perhaps resulting in reduced physical function and quality of life.

In the present study, frailty was predicted by physical function (6MWD and handgrip) and the number of exacerbations and comorbidities. This aligns with previous studies where the nonlinear association of frailty with age, functional dependence, and chronic disease has been established.^3,4,6,12–14,30^ Although there are few specific studies in individual chronic diseases, such as reported here, in the Women’s Health and Aging Studies, the risk of frailty increased with inflammatory comorbidities. This showed that the combination of pulmonary disease with anemia carried a risk ratio of 5.57 compared with comparator participants who had fewer comorbidities.^31^ A further study in this female cohort demonstrated that frailty was related nonlinearly to the number of abnormal physiological systems, which resembles the impact of multiple comorbidities in COPD.^12^ Such physiological deficits included loss of skeletal muscle mass (sarcopenia) and function, with progressive loss of capacity for physical activity, loss of bone mineral density, increased systemic inflammation, reduction in HRQoL, and increased cardiovascular morbidity and mortality.^22,32,33^

Our patients with COPD were stable at the time of recruitment but had higher levels of inflammation (HsCRP and fibrinogen) than comparators; however, the difference in fibrinogen between patients and comparators was small. The higher, than expected, levels of inflammation in comparators may be attributed to the age of the participants and potential presence of subclinical disease in some comparators despite being free from occult respiratory, cardiovascular disease, or inflammatory conditions.^34^ COPD is characterized by low-grade inflammation; both CRP and fibrinogen are acute phase proteins produced by the liver and have been associated with disease severity in COPD. However, fibrinogen is thought to be a more specific marker and has being associated with increased risk of exacerbation, hospitalization, and extra pulmonary manifestations.^35^ The reported associations with frailty and aging, in many ways parallels findings in COPD, where systemic inflammation may be causal, compensatory, or an epi-phenomenon.

This accumulation of deficits in COPD resembled the pattern of that occurring with aging healthy populations, potentially allowing the prediction of poor outcomes such as increased risk of falls, hospitalization, residential care, reduced HRQoL, disability, and increased mortality.^34,6,12–14,30^ Although there are a number of ways of determining frailty, the CGA has the advantage of being a simple, rapid questionnaire that is applicable for all health-care sectors.

### Limitations of the study

As with the majority of cross-sectional studies, a limitation of the findings is that they may be influenced by bias, confounding, and lack of causality. Although patients and comparators were similar, they were not matched and there were differences in the number of comorbidities between the groups. This, however, reflects the multisystem nature of the COPD inherent to the disease. The ARCADE study was designed to describe a representative COPD group and a comparator group rather than a true controlled study. Frailty did not relate to age and after adjustment for smoking history, frailty remained elevated in COPD in line with Park’s study where smoking was unrelated to frailty.^10^ A limitation of the CGA questionnaire to determine frailty is that it relies on patient recall; nevertheless, many of the parameters could be verified by medical notes, physical assessment, and carers.

## Conclusion

Patients with COPD were frailer than a comparator group of current or ex-smokers, independent of age. Frailty was predicted by the number of comorbidities and the number of exacerbations per year as well as physical function. This study highlights the need to take a global view of COPD to quantify the systemic nature of the disease. The CGA is a quick, simple, and inexpensive questionnaire that provides a global assessment of COPD. The CGA may be a useful clinical tool to highlight the multisystem deficits of COPD, which could guide interventions, monitor disease processes, and improve patient care.

## Supplemental material

Supplemental Material, CGA_questionairre - Frailty: A global measure of the multisystem impact of COPD Click here for additional data file. Supplemental Material, CGA_questionairre for Frailty: A global measure of the multisystem impact of COPD by Nichola S Gale, Ali M Albarrati, Margaret M Munnery, Ruth E Hubbard, Ruth Tal-Singer, John R Cockcroft, and Dennis J Shale in Chronic Respiratory Disease

## References

[1] Divo M Cote C de Torres J Comorbidities and risk of mortality in patients with chronic obstructive pulmonary disease. Am J Respir Crit Care Med 2012; 186: 155–161. 22561964 10.1164/rccm.201201-0034OC

[2] Vanfleteren L Spruit MA Groenen M Clusters of comorbidities based on validated objective measurements and systemic inflammation in patients with chronic obstructive pulmonary disease. Am J Respir Crit Care Med 2013; 187: 728–735. 23392440 10.1164/rccm.201209-1665OC

[3] Fried LP Tangen CM Walston J Frailty in older adults: evidence for a phenotype. J Gerontol A Biol Sci Med Sci 2001; 56: M146–M157. 11253156 10.1093/gerona/56.3.m146

[4] Rockwood K Mitnitski A Frailty defined by deficit accumulation and geriatric medicine defined by frailty. Clin Geriatr Med 2011; 27: 17–26. 21093719 10.1016/j.cger.2010.08.008

[5] Clegg A Young J Iliffe S Frailty in elderly people. Lancet 2013; 381: 752–762. 23395245 10.1016/S0140-6736(12)62167-9 PMC4098658

[6] Romero-Ortuno R Kenny RA The frailty index in Europeans: association with age and mortality. Age Ageing 2012; 41: 684–689. 22522775 10.1093/ageing/afs051 PMC3424051

[7] Galizia G Cacciatore F Testa G Role of clinical frailty on long-term mortality of elderly subjects with and without chronic obstructive pulmonary disease. Aging Clin Exp Res 2011; 23: 118–125. 21743290 10.1007/BF03351076

[8] Maddocks M Kon SSC Canavan JL Physical frailty and pulmonary rehabilitation in COPD: a prospective cohort study. Thorax 2016; 71(11): 988–995. 27293209 10.1136/thoraxjnl-2016-208460 PMC5099190

[9] Lahousse L Maes B Ziere G Adverse outcomes of frailty in the elderly: the Rotterdam study. Eur J Epidemiol 2014; 29: 419–427. 24935872 10.1007/s10654-014-9924-1

[10] Park SK Richardson CR Holleman RG Frailty in people with COPD, using the National Health and Nutrition Evaluation Survey dataset (2003–2006). Heart Lung 2013; 42: 163–170. 23535142 10.1016/j.hrtlng.2012.07.004 PMC4020241

[11] Kusunose M Oga T Nakamura S Frailty and patient-reported outcomes in subjects with chronic obstructive pulmonary disease: are they independent entities? BMJ Open Respir Res 2017; 4: e000196. 10.1136/bmjresp-2017-000196 PMC5531303 28883929

[12] Fried LP Xue QL Cappola AR Nonlinear multisystem physiological dysregulation associated with frailty in older women: implications for etiology and treatment. J Gerontol A Biol Sci Med Sci 2009; 64: 1049–1057. 19567825 10.1093/gerona/glp076 PMC2737590

[13] Rockwood K Song X MacKnight C A global clinical measure of fitness and frailty in elderly people. CMAJ 2005; 173: 489–495. 16129869 10.1503/cmaj.050051 PMC1188185

[14] Jones D Song X Mitnitski A Evaluation of a frailty index based on a comprehensive geriatric assessment in a population based study of elderly Canadians. Aging Clin Exp Res 2005; 17: 465–471. 16485864 10.1007/BF03327413

[15] Gale NS Albarrati AM Munnery MM Assessment of risk in chronic airways disease evaluation (ARCADE) protocol and preliminary data. Chron Respir Dis 2014; 11: 199–207. 25159833 10.1177/1479972314546765

[16] Hubbard RE Story DA Patient frailty: the elephant in the operating room. Anaesthesia. 2014; 69: 26–34. 24303858 10.1111/anae.12490

[17] Jones P Quirk F Baveystock C A self-complete measure of health status for chronic airflow limitation. The St. George’s respiratory questionnaire. Am Rev Respir Dis 1992; 145: 1321–1327. 1595997 10.1164/ajrccm/145.6.1321

[18] Jones PW Brusselle G Dal Negro RW Properties of the COPD assessment test in a cross-sectional European study. Eur Respir J 2011; 38: 29–35. 21565915 10.1183/09031936.00177210

[19] Global Initiative for Chronic Obstructive Lung Disease G. Global Strategy for the Diagnosis, Management and Prevention of COPD, Global Initiative for Chronic Obstructive Lung Disease (GOLD), 2015 http://www.goldcopd.org (accessed 9 April 2016).

[20] American Thoracic Society. ATS statement: guidelines for the six-minute walk test. Am J Respir Crit Care Med 2002; 166: 111–117. 12091180 10.1164/ajrccm.166.1.at1102

[21] Romero-Ortuno R An alternative method for frailty index cut-off points to define frailty categories. Eur Geriatr Med 2013; 4: 299–303. 10.1016/j.eurger.2013.06.005 PMC3873779 24379896

[22] Rossi AP Watson NL Newman AB Effects of body composition and adipose tissue distribution on respiratory function in elderly men and women: the health, aging, and body composition study. J Gerontol A Biol Sci Med Sci 2011; 66A: 801–808. 10.1093/gerona/glr059 PMC3143349 21498841

[23] Hubbard RE Lang IA Llewellyn DJ Frailty, body mass index, and abdominal obesity in older people. J Gerontol A Biol Sci Med Sci 2009; 65: 377–381. 19942592 10.1093/gerona/glp186

[24] Hubbard RE Rockwood K Frailty in older women. Maturitas 2011; 69: 203–207. 21570783 10.1016/j.maturitas.2011.04.006

[25] Lahousse L Ziere G Verlinden VJ Risk of frailty in elderly with COPD: a population-based study. J Gerontol A Biol Sci Med Sci 2016; 71: 689–695. 26355016 10.1093/gerona/glv154

[26] Divo MJ Casanova C Marin JM Chronic obstructive pulmonary disease comorbidities network. Eur Respir J 2015; 46: 640–650. 26160874 10.1183/09031936.00171614

[27] Cesari M Leeuwenburgh C Lauretani F Frailty syndrome and skeletal muscle: results from the Invecchiare in Chianti study. Am J Clin Nutr 2006; 83: 1142–1148. 16685058 10.1093/ajcn/83.5.1142 PMC2668161

[28] Walston J McBurnie M Newman A Frailty and activation of the inflammation and coagulation systems with and without clinical comorbidities: results from the cardiovascular health study. Arch Intern Med 2002; 162: 2333–2341. 12418947 10.1001/archinte.162.20.2333

[29] Angleman SB Harris TB Melzer D The role of waist circumference in predicting disability in periretirement age adults. Int J Obes Relat Metab Disord 2005; 30: 364–373. 10.1038/sj.ijo.0803130 16231023

[30] Rockwood K Mitnitski A Frailty in relation to the accumulation of deficits. J Gerontol A Biol Sci Med Sci 2007; 62: 722–727. 17634318 10.1093/gerona/62.7.722

[31] Chang SS Weiss CO Xue QL Patterns of comorbid inflammatory diseases in frail older women: the women’s health and aging studies I and II. J Gerontol A Biol Sci Med Sci 2010; 65A: 407–413. 10.1093/gerona/glp181 PMC3004772 19933749

[32] Bolton CE Ionescu A Shiels K Associated loss of fat-free mass and bone mineral density in chronic obstructive pulmonary disease. Am J Respir Crit Care Med 2004; 170: 1286–1293. 15374843 10.1164/rccm.200406-754OC

[33] Van Eeden S Leipsic J Paul Man SF The relationship between lung inflammation and cardiovascular disease. Am J Respir Crit Care Med 2012; 186: 11–16. 22538803 10.1164/rccm.201203-0455PP

[34] Miller BE Tal-Singer R Rennard SI Plasma fibrinogen qualification as a drug development tool in chronic obstructive pulmonary disease. Perspective of the chronic obstructive pulmonary disease biomarker qualification consortium. Am J Respir Crit Care Med 2016; 193: 607–613. 26745765 10.1164/rccm.201509-1722PP

[35] Miller J Edwards LD Agustí A Comorbidity, systemic inflammation and outcomes in the ECLIPSE cohort. Respir Med 2013; 107: 1376–1384. 23791463 10.1016/j.rmed.2013.05.001

