# Supplementary material for: Frailty: A global measure of the multisystem impact of COPD
Source: Chron Respir Dis. 2018 Jan 16;15(4):347–55. doi: 10.1177/1479972317752763 (PMC6234567; doi:10.1177/1479972317752763)
Supplement: Supplemental Material, CGA_questionairre - Frailty: A global measure of the multisystem impact of COPD [file CGA_questionairre.pdf]

Suppliment 1

# CGA-SF
# Comprehensive Geriatric Assessment Short Form

Patient ID

Completed by ................................... Date .................

| | | | | | | | |
|---|---|---|---|---|---|---|---|
| **Motivation** | **circle one:** | High | Usual | Low | | | |
| **Self rated health** | **circle one:** | Excellent | Good | Fair | Poor | Couldn't' say | |
| **Cognition** | **circle one:** | Normal | Mild cognitive impairment | Dementia | | | |
| | **circle all that apply:** | Agitation/wandering | Delusions/hallucinations | Delirium | | | |
| **Emotional** | Normal OR **circle all that apply:** | Anxiety | Bereavement | Depression | Fatigue | | |
| **Sleep** | Normal OR **circle all that apply:** | Poor or disrupted | Daytime drowsiness | | | | |
| **Communication** | **circle one for each** | | | | | | |
| | **Speech** Normal Impaired | **Hearing** Normal Impaired | **Vision** Normal Impaired | | | | |
| **Strength** | **Grip strength** Normal Weak | **Proximal muscle strength** Normal Weak | | | | | |
| | **Hemiparesis:** | **Arm** Yes No | **Leg** Yes No | | | | |

| | | | | | |
|---|---|---|---|---|---|
| **Mobility** | Transfer | Ind | Asst | Dep | |
| | Walking | Ind | Asst | Dep | **Slow ?** Yes No |
| | Aid used? | Walking stick | Frame | | |
| **Balance** | Balance | Normal | Impaired | | |
| | Falls | None | Yes | | |
| **Elimination** | Bowel | Cont | Occas accident | Incont | |
| | Bladder | Cont | Occas accident | Incont | |
| **Nutrition** | Weight change | Stable | Loss | Gain | |
| | Appetite | Normal | Fair | Poor | |
| | Weight | Normal | Under | Obese | |
| **ADLs** | Feeding | Ind | Asst | Dep | |
| | Bathing | Ind | Asst | Dep | |
| | Dressing | Ind | Asst | Dep | |
| | Toileting | Ind | Asst | Dep | |
| **IADLs** | Cooking | Ind | Asst | Dep | |
| | Cleaning | Ind | Asst | Dep | |
| | Shopping | Ind | Asst | Dep | |
| | Medications | Ind | Asst | Dep | |
| | Driving | Ind | Asst | Dep | |
| | Banking | Ind | Asst | Dep | |

**Social Engagement**

☐ Frequent
☐ Occasional
☐ Rarely

**Medical history**

☐ Hypertension
☐ COPD
☐ TIA/Stroke
☐ Angina/MI
☐ CHF
☐ Diabetes
☐ Cancer
☐ Alcohol excess
☐ Pressure sores
☐ Hip Fracture
☐ OA/RA
☐ Osteoporosis
☐ Parkinson's dis.

| Problems |
|---|
| 1. |
| 2. |
| 3. |
| 4. |
| 5. |
| 6. |
| 7. |

| Current medication |
|---|
| |

**Notes:**
